# Ngn2-Induced Differentiation of the NG108-15 Cell Line Enhances Motor Neuronal Differentiation and Neuromuscular Junction Formation

**DOI:** 10.3390/biom15050637

**Published:** 2025-04-29

**Authors:** Madeline Meli, Kristy Swiderski, Jinchao Gu, Ben Rollo, Ben Bartlett, Marissa K. Caldow, Gordon S. Lynch, Patrick Kwan, Huseyin Sumer, Brett A. Cromer

**Affiliations:** 1 Department of Chemistry and Biotechnology, School of Science, Computing and Engineering Technologies, Swinburne University of Technology, Melbourne, VIC 3122, Australia; mmeli@swin.edu.au (M.M.); 2 Centre for Muscle Research, Department of Anatomy and Physiology, School of Biomedical Sciences, Faculty of Medicine, Dentistry and Health Sciences, The University of Melbourne, Melbourne, VIC 3122, Australia; kristys@unimelb.edu.au (K.S.); marissa.caldow@unimelb.edu.au (M.K.C.); gsl@unimelb.edu.au (G.S.L.); 3 Department of Neuroscience, Central Clinical School, Monash University, Alfred Centre, Melbourne, VIC 3122, Australia; ben.rollo@monash.edu (B.R.); patrick.kwan@monash.edu (P.K.)

**Keywords:** neurons, cell line, motor neurons, muscle fibres, skeletal muscle, transcription factors, cell differentiation, myogenesis, neuromuscular junction, neuroblastoma

## Abstract

The neuronal progenitor NG108-15 neuroblastoma x glioma cell line proliferates indefinitely in vitro and is capable of directed differentiation into cholinergic neurons. The cell line is a robust model for investigating neuronal differentiation and function in vitro. The lineage-specific transcription factor-mediated differentiation of pluripotent stem cell lines (PSCs) leads to more rapid, efficient, and functional neurons. In this study, we tested the hypothesis that transcription factors could also drive the fate of an immortalised cell line. We first established a stable NG108-15 cell line, by piggyBac (pBac) transposition, that conditionally expresses neurogenin-2 (Ngn2), a common transcription factor for specifying neuronal fate. Following doxycycline-induction of Ngn2, we observed more rapid and efficient differentiation, and improved neurite outgrowth and viability compared with the WT cell line. Moreover, when co-cultured with C2C12 mouse myotubes, the modified NG108-15 cells resulted in significantly larger acetylcholine receptor (AChR) aggregates, suggesting enhanced neuromuscular junction (NMJ) formation. These findings describe a novel methodology for differentiating NG108-15 cells more efficiently, to enhance the usefulness of the cell line as a motor neuron model.

## 1. Introduction

Neuromuscular junction (NMJ) impairments are known to be involved in various motor neuron diseases, such as spinal muscular atrophy, and autoimmune diseases, such as myasthenia gravis [1]. Research into these conditions can be enhanced by using model NMJs in vitro, via culture combinations of motor neurons and skeletal myotubes, which can be sourced from various origins. It important that in vitro models of motor neurons are robust, but also cost-effective and easy to culture.

Cells from various origins can be used for in vitro neuronal modelling, including primary-cultured neurons, stem cell-derived neurons and immortalised neuronal cell lines. While primary neuronal cell cultures are useful, as the cells are already in their native state, their application is limited as they show little or no replication in vitro. Stem cells and immortalised neuronal cell lines are useful alternatives as they can proliferate indefinitely in culture [2,3], and in vitro differentiation allows for neuronal development to be examined. For many decades, immortalised neuronal cell lines have served as robust in vitro neuronal models, where neuronal differentiation is generally technically straightforward, reliably generating relatively homogenous cultures of differentiated neurons [4]. The availability of pluripotent stem cells (PSCs) enabled alternative approaches, with initial methods for neuronal differentiation involving lengthy stepwise medium changes through precursors mediated by growth factors and supplements, in a process termed “directed differentiation” [5]. More recent developments have demonstrated that induced ectopic expression of specific transcription factors (TFs) can rapidly and efficiently drive neuronal differentiation in PSCs, in a process termed “neuronal induction” [5].

One such transcription factor, Ngn2, belonging to the neurogenin-2 family, encodes a basic helix-loop-helix (bHLH) protein involved in directing neuronal fate in neurogenesis. Thoma and colleagues (2012) showed that ectopic expression of Ngn2 in mouse ES cells via Tol2 plasmid transposition was effective at neuronal induction, giving rise to a high percentage of glutamatergic neurons expressing ßIII tubulin [6]. Whole-cell patch-clamp recordings showed that the induced neurons had robust action potentials (APs) within 10 days that were able to form synapses with co-cultured primary neurons, highlighting the key role of Ngn2 within early mammalian neuronal development [6]. Later work by Zhang et al. (2013) [7] extended this concept into hiPSCs, where they induced glutamatergic neurons with a yield of almost 100% within 2 weeks of lentiviral delivery of Ngn2. The induced neurons presented a mature morphology as demonstrated by the formation of mature synapses, and successful integration of synaptic networks when transplanted into a mouse brain. Additionally, the molecular properties of the induced glutamatergic neurons confirmed the expression of AMPA-type glutamate receptors, including GluA1, A2, and A4. Nearly all the induced neurons expressed vesicular glutamate transporter vGlut2, with some cells expressing vGlut1 (20%) as well as a high expression of GABA_A_ receptors. Conversely, the vesicular GABA transporter vGAT was not expressed. Electrophysiology confirmed the induced neurons’ ability to form APs, a defining property of mature neurons, including voltage-gated Na^+^ and K^+^ currents, which were comparable in both ESC and iPSC-derived cell lines. Ngn2-induced differentiation is now a well-established tool used to rapidly induce functional neurons from PSCs or fibroblasts [8]. Furthermore, transcription factor-mediated differentiation can give rise to several mature neuronal lineages such as glutamatergic [6,7,9], cholinergic [10], dopaminergic [11], motor [12], and sensory neurons [13].

Although PSC-derived induced neurons are well-established for producing mature neurons in vitro, immortalised neuronal cell lines remain a widely used alternative, as they offer simple, robust, and reproducible cell cultures. For example, the NG108-15 cell line is a hybrid of mouse N18TG2 neuroblastoma cells and rat C6BU-1 glioma cells [14]. Once NG108-15 cells enter the differentiation process, they adopt phenotypic properties such as neurite elongation and the expression of choline acetyltransferase (ChAT), which produces acetylcholine. Once differentiated, the cell line closely mimics cholinergic motor neurons. NG108-15 cells are advantageous because they are cost-effective, relatively easy to culture and are one of the most useful models of cholinergic neurons. Whitemarsh and colleagues investigated the sensitivity of various cell lines to botulinum neurotoxins (BoNTs), one of the most toxic substances known to humans [15]. BoNTs inhibit cholinergic neurotransmission at neuromuscular junctions but are known to enter other neurons [15,16]. They found that the NG108-15 cell line was the most capable of detecting BoNT type A subtype 1, compared to several other immortalised cell lines, such as PC12 and Neuro-2a cells, highlighting their usefulness as a cholinergic motor neuron model.

In this study, we investigate a hybrid model, combining the advantages of immortalised cell lines with those of TF-driven induced neurons. We test the hypothesis that the ectopic expression of Ngn2 provides a novel approach for improving the differentiation potential of the NG108-15 cell line. Stable integration of Ngn2 using a piggyBac vector and inducible expression with doxycycline improved the differentiation of NG108-15 cells while maintaining a cholinergic phenotype, enhancing neurite outgrowth, and improving cell viability compared with the WT cell line. Furthermore, when co-cultured with myotubes, the modified NG108-15 cells produced significantly larger AChR aggregates, indicating enhanced NMJ formation. These findings enhance the applicability of the NG108-15 cell line as a robust in vitro neuronal model.

## 2. Materials and Methods

### 2.1. NG108-15 Cell Culture and Differentiation

This study used cell culture and genetic modification to assess the effects of Ngn2 on differentiation of the NG108-15 cell line (sourced from ATCC). This study was conducted at Swinburne University of Technology and The University of Melbourne ethics number 2016SBC03. NG108-15 cells were grown and maintained in DMEM supplemented with 10% foetal bovine serum (FBS) and 0.5% pen/strep (ThermoFisher Scientific, Mulgrave, VIC, Australia), and subcultured when it was approximately 70–80% confluent at a 1:5 ratio in T25 or T75 flasks and incubated at 37 °C with 5% CO_2_ overnight. Differentiation of NG108-15 cells results in decreased proliferation and morphological changes, including neurite elongation, characterised by increasingly long and thin arborisations [17]. For neuronal differentiation, NG108-15 cells were seeded at 2 × 10^4^/cm^2^ in 24 or 6-well plates in growth medium. For immunocytochemistry, cells were seeded in chambered cover glass slides coated with poly-l-ornithine followed by laminin (10 µg/mL in PBS), suspended in a Grace Bio-Labs ProPlate tray (Sigma Aldrich, Castle Hill, NSW, Australia). For neuronal differentiation, the medium was replaced with Neurobasal medium containing neuronal supplements N2 (1%) and B27 (2%) (ThermoFisher Scientific, Mulgrave, VIC, Australia), 0.5% pen/strep, 0.5% FBS and doxycycline (1 µg/mL) (Sigma Aldrich, Castle Hill, NSW, Australia) for Ngn2-induced differentiation. Doxycycline was included in the medium to induce gene expression for the entire differentiation period. The WT and Ngn2-induced NG108-15 cells were both differentiated in the same medium. For viability assays, FBS was excluded from the differentiation medium.

### 2.2. C2C12 Cell Culture

The immortalised mouse myoblast C2C12 cell line (ATCC, Manassas, VA, USA) was primarily co-cultured with differentiating NG108-15 cells. Cells were grown in DMEM supplemented with 10% FBS. C2C12 cells were grown and maintained in 10 cm Standard Tissue Culture Dishes or T25 flasks in 5 mL of growth medium. When the cells were 50–60% confluent, the cells were detached using Trypsin-EDTA (0.25%), or TrypLE (ThermoFisher Scientific, Mulgrave, VIC, Australia) and incubated at 37 °C with 5% CO_2_ for 2–3 min. The cells were then seeded at a 1:5 ratio and returned to the incubator at 37 °C with 5% CO_2_.

### 2.3. Co-Culture of C2C12 and NG108-15 Cells

C2C12 cells differentiate into multinucleated myotubes by the fusion of undifferentiated mononucleated C2C12 cells. For muscle/neuronal co-culture, the C2C12 cells were seeded at 2.8 × 10^4^ cells per cm^2^ in ibiTreat µ-Slide chambered coverslips (ibidi, Gräfelfing, Germany) or 1 × 10^5^ in 12-well plates and were grown to 90–95% confluency. The growth medium was replaced with myotube differentiation medium DMEM and 2% horse serum (ThermoFisher Scientific, Mulgrave, VIC, Australia) to mature myotubes for 4 days. On day 4, NG108-15 cells were detached and centrifuged, and the cell pellet was resuspended in the neuronal differentiation medium. The myotube differentiation medium was removed and replaced with neuronal differentiation medium, and the neuronal cells were seeded on top of the myotubes at 2 × 10^4^ cells per cm^2^ in ibiTreat µ-Slide chambered coverslips or 8 × 10^4^ in 12-well plates. The co-cultures were maintained for 7 days in neuronal differentiation medium before performing experiments, with medium changes every 2 days.

### 2.4. Genetic Modification of NG108-15 Cells

NG108-15 cells were stably transfected using the pBac/Ngn2 plasmid for transcription factor-induced differentiation. The pBac_Ngn2 plasmid was produced using pTet-O-Ngn2-puro, a gift from Marius Wernig (Addgene plasmid # 52047; http://n2t.net/addgene:52047; RRID: Addgene_52047), accessed on 25 April 2025 [7]. Lipofection was performed using Lipofectamine™ 2000 according to the manufacturer’s instructions (ThermoFisher Scientific, Mulgrave, VIC, Australia). NG108-15 cells were seeded at 4 × 10^4^ cells per well in 24-well plates, and the cells were incubated at 5% CO_2_ 37 °C. The following day, cells were transfected with the pBac_Ngn2 plasmid. Tube A contained lipofectamine (1 µL) in 25 µL of Opti-MEM (ThermoFisher Scientific, Mulgrave, VIC, Australia). Tube B contained pBac_Ngn2 DNA (600 µg) and super pBac transposase (200 µg) in 25 µL of Opti-MEM. Tubes A and B were then combined to form the DNA/lipid complex, which was added to cells, which was then incubated at 37 °C with 5% CO_2_ overnight. To select for stable incorporation, 1–2 µg/mL of puromycin (InvivoGen, San Diego, CA, USA) was added to the cells for up to 7 days.

### 2.5. Fluorescence Activated Cell Sorting (FACS)

NG108-15 cells were sorted using FACSAria III (BD FACSAria III software). This was performed to obtain cell lines with specified fluorescent protein expression levels to select for various levels of Ngn2 expression. To prepare, the cells were grown to confluency. The cells were then detached and resuspended in growth medium and centrifuged at 1200× *rpm* for 5 min. The cells were then resuspended in 1 mL of FACS buffer (PBS with 1% FBS). The cell suspension was then filtered into a 5 mL round bottom Falcon tube with a filter lid, and the tubes were stored in ice. During sorting, the cells were collected using 5 mL round bottom Falcon tubes containing 1 mL of FACS buffer, the cell suspension was transferred to a 15 mL Falcon tube and the cells were centrifuged at 1200× *rpm* for 5 min to remove the FACS buffer. The cell pellet was then resuspended in growth medium and expanded.

### 2.6. Viability Assay

A viability assay was performed to investigate the viability of NG108-15 cells following induced-differentiation of pBac_Ngn2 compared to the WT cells. NG108-15 cells were stained with Hoechst 33342 to label all cells and Propidium Iodide (PI) to label dead cells (Sigma Aldrich, Castle Hill, NSW, Australia). The cells were stained with 100 µL of the Hoechst 33342 solution (20 µg/mL in PBS) and incubated for 15 min at 37 °C with 5% CO_2_. To label dead cells, PI (5 µg/mL) was added to the wells and left for 5 min at room temperature. Cells were imaged in random areas 5 times for each group using an epifluorescence microscope (Nikon Eclipse Ti Epifluorescence Microscope) (Nikon corporation, Tokyo, Japan). The images were then opened using ImageJ software (version 1.54h, Java 1.8.0_172 (64-bit), and all cells and dead cells were manually counted using the multi-point tool, and the percentage of viable cells quantified.

### 2.7. Immunofluorescence

Immunofluorescence assays were performed for the molecular characterisation of NG108-15 cells, which were stably transfected with pBac_Ngn2 compared to the WT and undifferentiated cells. For fixing, the medium was aspirated and 4% paraformaldehyde (PFA) (Santa Cruz Biotechnology, Dallas, TX, USA) in PBS was added to cover the wells, which was incubated for 15 min. After fixing, the PFA solution was removed, and the cells were washed 3 times with PBS for 5 min. After fixing, the cells were permeabilised and stained simultaneously in a blocking solution containing 1% goat serum, 0.1% Triton × 100 (ThermoFisher, Mulgrave, VIC, Australia), in PBS containing the diluted primary antibodies. The cells were then incubated for 1 h at 37 °C or overnight at 2–8 °C. After incubation, the cells were washed with PBS 3 times for 5 min each and the secondary antibodies (diluted in the blocking solution) were added to the wells and incubated in the dark for 1 h at 37 °C or overnight at 2–8 °C. Following incubation, the wells were washed with PBS 3 times for 5 min each. To label nuclei, cells were stained with Hoechst 33342 in PBS (20 µg/mL) in the dark for 15 min at 37 °C. Following incubation, the cells grown on glass slides were mounted with a drop of ProLong™ Gold Antifade Mountant (ThermoFisher Scientific, Mulgrave, VIC, Australia) and sealed with nail varnish. For cells grown in ibiTreat µ-Slide chambered coverslips, PBS was added to cover the wells. The cells were imaged using a confocal microscope (Olympus FV300 Laser Confocal Scanning Microscope, Hamburg, Germany).

### 2.8. Immunocytochemistry Reagents

The primary antibodies used for immunofluorescence were the following: anti-ßIII tubulin (1:500, previously Covance mms-435p) (BioLegend, San Diego, CA, USA), anti-Synapsin I (1:500, ab64581) and anti-choline acetyltransferase (ChAT) (1:500, ab181023) (Abcam, Melbourne, VIC, Australia), anti-vesicular glutamate transporter 1 (vGlut1) (1:500, 821301) (BioLegend, San Diego, CA, USA), anti-microtubule associated protein 2 (MAP2) (1:500, sc-32791) (Santa Cruz Biotechnology, Dallas, TX, USA). The secondary antibodies used for immunostaining were the following: Alexa Fluor 633 conjugated anti-mouse IgG H&L (1:500, a-21050) (ThermoFisher Scientific, Mulgrave, VIC, Australia). Alexa Fluor 594 conjugated anti-mouse IgG (H&L) (1:500, ab150116) (Abcam, Melbourne, VIC, Australia) and Alexa Fluor 647 conjugated anti-rabbit IgG H&L (1:500, ab150083) (Abcam, Melbourne, VIC, Australia), Alexa Fluor 488 anti-chicken IgY H&L (1:500, A-32931) (ThermoFisher Scientific, Mulgrave, VIC, Australia) and Alexa Fluor 647 anti-mouse IgG H&L (1:500, A32728) (ThermoFisher Scientific, Mulgrave, VIC, Australia).

### 2.9. Neurite Quantification

Neurite quantification was performed as an indicator of the morphology of the differentiated cell lines. The average neurite length per cell was quantified using the automatic neurite quantification plugin NeurphologyJ on ImageJ [18]. After differentiation, the neuronal cultures were stained with ßIII tubulin to label neuronal processes and Hoechst 33342 to label nuclei. The samples were then imaged 5 times in random areas using confocal microscopy. For image processing, the images labelling neuronal processes were isolated, and the ‘Subtract Background’ tool was applied using a rolling ball radius of 50 px. The ‘Gaussian Blur’ tool was then selected using a radius of 2. Neurite length was quantified by opening the NeurphologyJ interactive plugin, and the instructions were followed. The total neurite length was then divided by the number of nuclei to calculate the average neurite length per cell, which was converted into microns.

### 2.10. Quantification of Acetylcholine Receptor Clusters in Myotubes

C2C12-derived myoblasts are known to form NMJ-like formations when co-cultured with several motor neuron-like cell lines, including NG108-15 cells [19], so they were chosen to investigate the functional capacity of modified NG108-15 cells. The C2C12 cells were differentiated into myotubes in ibiTreat µ-Slide chambered coverslips with and without NG108-15 co-culture. For immunostaining, the AChRs were labelled using an Alexa Fluor 647 fluorescent conjugated α-bungarotoxin (1:500, B35450) (Invitrogen, ThermoFisher Scientific, Mulgrave, VIC, Australia). The cultures were imaged 10 times in random areas in each well using a confocal microscope. The images containing the AChRs were isolated using ImageJ, the aggregates were isolated using the ‘Threshold’ tool, and the ‘Analyse Particles’ tool was selected to display the measurements. The average size of AChR clusters was quantified by dividing the total area of the clusters (um^2^) by the number of clusters.

### 2.11. mRNA Extraction, and mRNA Analysis

mRNA sequencing of differentiated modified and unmodified NG108-15 cells was performed to investigate and quantify the expression of a wide array of genes. RNA extractions were performed using the RNeasy^®^ Mini Kit (QIAGEN, Clayton, VIC, Australia), which was sent to the Beijing Genomic Institute (BGI) for RNA Transcriptome sequencing (DNBSEQ G-400 platform) using 150FE of the DNBSEQ platform (BGI Genomics, Shenzhen, China). To prepare, cells were seeded in 6-well plates at 2 × 10^5^ cells per well. Cell samples were collected after 7 days of differentiation or the day after seeding for the undifferentiated controls. RNA was purified from cell samples according to the manufacturer’s instructions (RNeasy^®^ Mini Handbook, QIAGEN) [20]. Three biological replicates were prepared for each sample, which had an RNA integrity number > 8.0 and a level A quality, as provided in the BGI sample testing report. For analysis, the counts per million (CPM) were displayed directly in bar charts, in Venn diagrams using Venny [21] and in a Multidimensional scaling (MDS) plot using Degust (Monash University, Melbourne, Australia). Log2fold change (log2FC) values were displayed in an expression-based heatmap using Heatmapper [22] and a volcano plot using VolcaNoseR [23] to provide an overview of several cholinergic and neuronal genes. Heatmaps were generated for key neuronal genes, as well as all genes which were clustered according to average linkage. To generate the volcano plot, the log2FC is plotted for each gene in the Ngn2-L differentiated cells relative to WT differentiated cells, according to the CPM value, with statistical significance on the *y*-axis. To generate the heatmap, the log2FC of genes in WT and Ngn2-L cell lines was quantified relative to the undifferentiated control. The log2FC was quantified using the CPM value for each replicate, relative to the average CPM of the undifferentiated control.

### 2.12. Quantification of Myotube Contraction

C2C12-derived myotubes are capable of spontaneous contraction due to the expression of contractile proteins throughout differentiation [24]. Several systems can be used for quantifying the frequency of muscle cell contraction in vitro, which typically requires electrophysiological equipment, which can be time-consuming and difficult to access. Conversely, muscle contraction can be easily and non-invasively measured using time-series (video) microscopy to provide kymographs, providing an alternative to electrophysiological methods [25].

Spontaneous myotube contraction dynamics were quantified in myotube/neuronal co-cultures or single-cell myotube cultures after 11 days of differentiation. Between 5 and 10 contracting myotubes were imaged at 10× magnification for each group. To perform time-series imaging, the manual for the microscope (EVOS™ M5000 Imaging System) was followed. The contractions were recorded at 1 fps (frame per second) for 200 s to generate time-series images. The time-series images were analysed using ImageJ using the KymoResliceWide plugin. For processing, the image was smoothed using Process > Smooth. To generate the kymographs, a line was drawn from the outside to the inside of the myotube using the line tool, and ‘KymoResliceWide’ was selected. A vertical line was then drawn down the resliced image, and Analyse > Plot Profile was selected. Once the plot profile was generated, a pixel intensity baseline was chosen to reduce background noise and random artefacts. Contractions (peaks) below the predetermined baseline were excluded. The contraction frequency, time taken to reach the peak, and time between peaks were quantified using the Spiky toolkit [26], originally designed for cardiomyocytes.

### 2.13. Graphs and Statistical Analysis

Graphs were produced using OriginPro 2020 (64-bit), SR1, Version 9.7.0.188. For statistical analysis, results are expressed as the mean ± SEM for experiments. Statistical significance was calculated using Microsoft Excel using a two-tailed *t*-test (unpaired). Statistical significance was determined at *p* < 0.05 (*), *p* < 0.01 (**), and *p* < 0.005 (***).

## 3. Results

### 3.1. Selecting for Various Gene Expression Levels in NG108-15/Ngn2 Cells According to Fluorescence

The NG108-15 cells were stably transfected with a piggyBac (pBac)_Ngn2 plasmid, together with a helper plasmid expressing the piggyBac transposase, which inserts DNA from the pBac plasmid into TTAA sites randomly distributed in the cell’s genome. The pBac_Ngn2 plasmid contains a tetracycline response element and minimal CMV promoter to drive doxycycline-inducible Ngn2 expression, along with constitutively expressed puromycin resistance GFP marker genes to select for stable chromosomal insertion [27]. pBac transposition involves high copy number insertions throughout the genome transposition using a “cut-and-paste” mechanism as the transposase will recognise ITRs on both ends of the pBac vector and then integrate its DNA into the ‘TTAA’ transposon sites [28].

**Figure 1:**
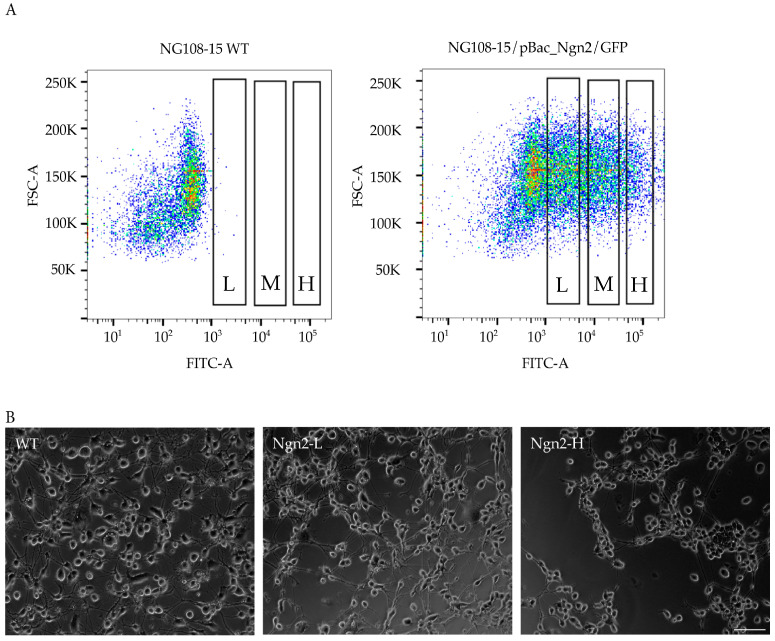
FACS and differentiation of NG108-15/Ngn2 cells. (**A**) FACS dot plot of GFP fluorescence intensity (*x*-axis) of NG108-15/pBac_Ngn2 cells against forward scatter (FSC-A, *y*-axis). pBac_Ngn2/GFP transfected NG108-15 cells were sorted according to fluorescence intensities, with gates represented by the black rectangles, from left to right: Low (L), Medium (M), and High (H). Data presented using FlowJo software (version 10.8.1; Tree Star, Inc., Ashland, OR, USA). (**B**) After 7 days of differentiation, the NG108-15/Ngn2-L (**middle**) and Ngn2-H cell lines (**right**) were morphologically similar, although there was enhanced neurite branching in the Ngn2-L cell line. Compared to the WT control (**left**), both the NG108-15/Ngn2-L and Ngn2-H cell lines appeared more morphologically homogenous. Magnification 10×. Scale bar 100 µm.

As these sites are randomly dispersed throughout the cell’s genome, the level of gene integration can vary and subsequently, gene expression levels may vary between transfected cells. It was anticipated that the fluorescence intensity of GFP-expressing cells would correlate with the number of insertions or copy numbers and the level of inducible Ngn2 expression, enabling flow cytometry to sort cells with different levels of Ngn2 expression [29,30,31]. To determine the optimal level of Ngn2 expression for neuronal differentiation, the stably transfected cells were sorted by GFP intensity into three cell pools (low, medium, and high). The WT NG108-15 cell line acted as a negative control, for gating of non-fluorescent cells, viability, morphology, and the selection of singlets, followed by the selection of cells according to low, medium, and high fluorescence intensities (Figure 1A). The relative fluorescence units (RFU) for each parameter after sorting corresponded to 10^3^, 10^4^ and 10^5^ respectively. The resulting cell pools isolated by FACS were labelled as Ngn2-L (low fluorescence), Ngn2-M (medium fluorescence), and Ngn2-H (high fluorescence).

Following induction of Ngn2 expression with doxycycline in the NG108-15/Ngn2-L, M and H cell lines, the cells were monitored every 2–3 days to investigate any effects on morphology or viability of the cell cultures. By day 7, there was no discernible difference in viability between the cell lines, indicating that Ngn2 expression is not deleterious to NG108-15 cells over a wide range of concentrations. Qualitatively, Ngn2-induced differentiation resulted in relatively mature neuronal features, such as neurites, compared with the WT control (Figure 1B). Surprisingly, the NG108-15 Ngn2-H cell line had fewer neurites and branching and an overall less mature neurite network compared with the Ngn2-L cell line (Figure 1B). Thus, there appears to be an optimal level of Ngn2 expression with higher levels being less effective in terms of neuronal morphology, so the Ngn2-L line was selected for future induced differentiation experiments.

### 3.2. Ngn2 Improves Neuronal Morphology and Retains a Cholinergic Phenotype Following Induced Differentiation

**Figure 2:**
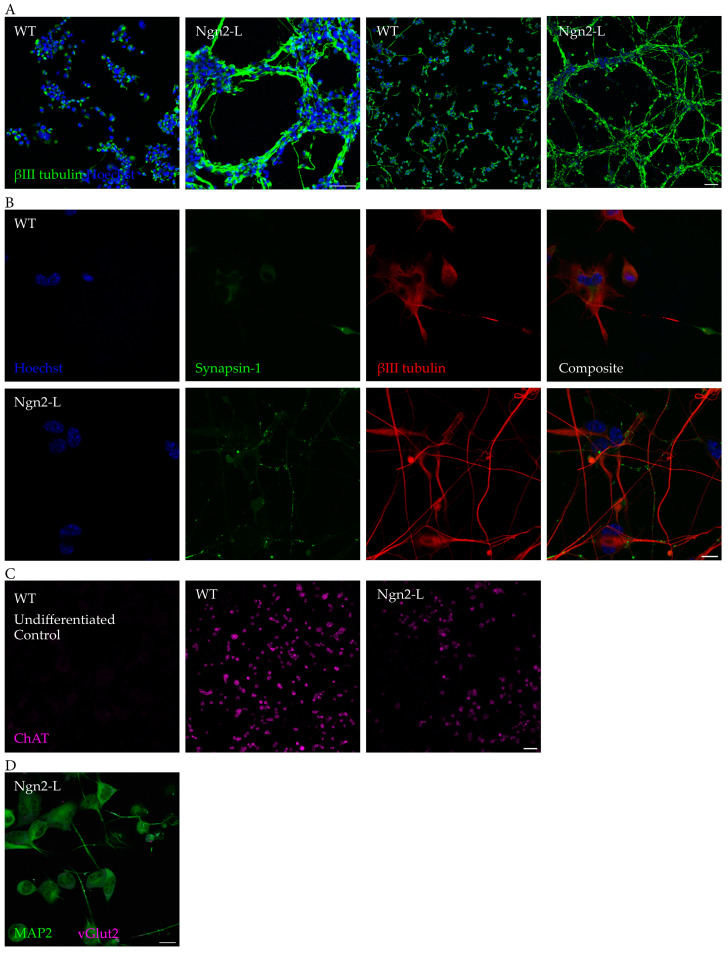
Neuronal marker expression in WT NG108-15 and NG108-15/Ngn2-L cells after 7–21 days of differentiation. (**A**) Confocal images of NG108-15 cells stained with the neuronal marker ßIII tubulin (green) and Hoechst 33342 (blue) 7 days after induced differentiation. WT NG108-15 cells (left) and the NG108-15/Ngn2-L cell line (right). Magnification 20× (left), 10× (right). Scale bar 100 µm. (**B**) Cells were stained with Syn1 (green), ßIII tubulin (red), and Hoechst (33342) (blue) 21 days after induced differentiation. There was little Syn1 expression in the NG108-15 WT control, but the Ngn2-L cells presented increased Syn1 immunoreactivity, showing as puncta along neurites/axons. Magnification 60×. Scale bar 20 µm. (**C**) ChAT immunoreactivity of NG108-15 WT and Ngn2-L cells after 7 days of differentiation, including an undifferentiated control, shows that both differentiated WT and Ngn2-L-induced cells had ChAT immunoreactivity (magenta). Magnification 10×. Scale bar 100 µm. (**D**) vGlut1 immunoreactivity of NG108-15/Ngn2-L cells after 7 days of differentiation shows that the Ngn2-L-induced cells did not have vGlut1 immunoreactivity (magenta). Magnification 10×. Scale bar 100 µm.

To further investigate the post-induction differentiation phenotype of NG108-15/Ngn2-L cells, compared to WT NG108-15 cells, these cells were stained with ßIII tubulin to label neuronal processes (Figure 2A,B). The induced NG108-15/Ngn2-L cells showed markedly stronger staining for ßIII tubulin and a more mature morphology with robust neurite elongation and branching compared to the WT control, 7 days post-induction. There is also prominent bundling of neurites in NG108-15/Ngn2-L cells, not apparent in WT cells (Figure 2A), another indication of a more mature neuronal phenotype.

To investigate the effects of Ngn2 on synapse formation, the NG108-15 cells were differentiated and stained after 21 days with the Synapsin I (Syn1) antibody. Syn1 is the most prevalent phosphoprotein within synapses, and it is involved with the clustering of synaptic vesicles to regulate NT release [32]. There were relatively low levels of Syn1 in the differentiating WT NG108-15 cultures (Figure 2B), which was expected given that differentiated NG108-15 cells typically express relatively low levels of synapsins [33]. There was, however, noticeably higher immunoreactivity of Syn1 in the NG108-15/Ngn2-L cells (Figure 2B) in prominent puncta, consistent with synaptic localisation.

Once NG108-15 cells reach a differentiated state, they begin to express ChAT, a marker of the cell line’s default cholinergic phenotype. To determine whether Ngn2-induced differentiation promotes ChAT-positive cells, differentiated cultures were stained for ChAT immunoreactivity. After differentiation, the NG108-15/Ngn2-L cells showed clear ChAT immunoreactivity (Figure 2C), indicating a cholinergic phenotype, like WT. To test whether the NG108-15/Ngn2-L cells expressed the vesicular glutamate transporter 1 (vGlut1), the cells were stained for vGlut1 immunoreactivity. After differentiation, the NG108-15/Ngn2-L cells were vGlut1-negative (Figure 2D). Thus, Ngn2-induced differentiation enhanced morphological and phenotypic markers of neuronal differentiation, whilst maintaining the direction of a cholinergic phenotype.

### 3.3. Ngn2-Induced Differentiation Improves the Neurite Length of Differentiating Cultures

**Figure 3:**
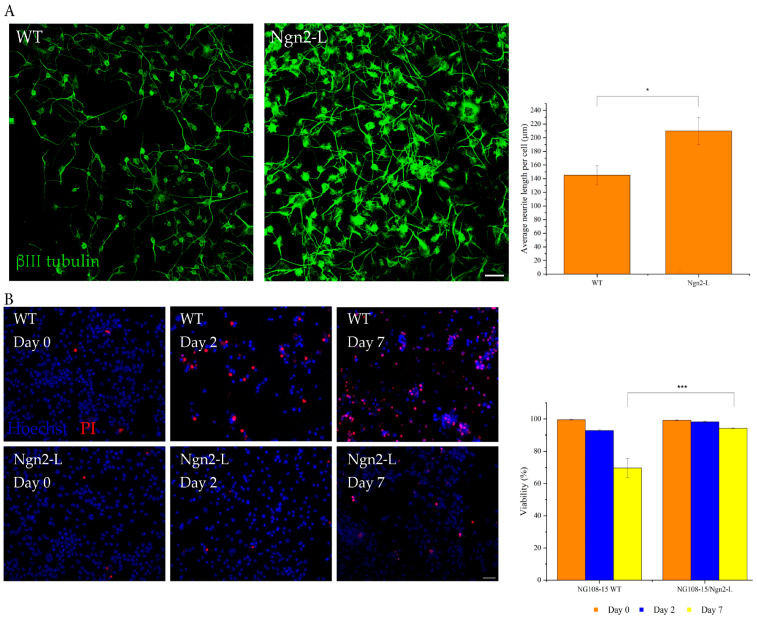
Effects of Ngn2-L-induced differentiation on NG108-15 cells according to neurite length and cell viability. (**A**) WT and Ngn2-L NG108-15 cells were differentiated for 4 days to investigate neurite morphology. Neuronal processes were labelled using ßIII tubulin immunofluorescence. The average neurite length per cell was significantly higher for Ngn2-L cells, * *p* = 0.0331. Results presented as mean ± SEM of *n* = 5 images analysed. Magnification 10× Scale bar 100 µm. (**B**) A viability assay was performed on NG108-15/Ngn2-L and WT control cells on days 0, 2, and 7 of differentiation, with all cell nuclei stained with Hoechst and dead cells stained with PI. By day 7, there were significantly fewer dead Ngn2-L cells than for the WT control. Results presented as means ± SEM, *n* = 5 images analysed. *** *p* = 0.0031. Magnification 10×. Scale bar 100 µm.

To evaluate neurite morphology, the average neurite length per cell was quantified for the WT and Ngn2-L cultures on day 4 of differentiation (Figure 3A). While the effects of Ngn2-L-induced differentiation were most noticeable when the cells were differentiated for at least 7 days, the complex morphology of the differentiated cells, including the bundling of neurites and clustering of nuclei (Figure 2A), made neurite quantification difficult. This is because single cells and neurites could not be accurately isolated using the software and resulted in inaccurate readings. To improve accuracy, the average neurite length was quantified on day 4 of differentiation rather than on day 7. The average neurite length for NG108-15/Ngn2-L cells was 210 ± 20 μm which was significantly longer (*p* = 0.0331) than for WT cells (145 ± 14 μm). These results indicate that Ngn2-induced differentiation enhances neurite complexity relative to the differentiation of WT NG108-15 cells.

### 3.4. Ngn2-Induced Differentiation Improves the Viability of Differentiating Cultures

A cell viability assay was conducted after 0, 2, and 7 days of differentiation to investigate whether Ngn2-induced differentiation affects the viability of the cell line. Here, FBS (0.5%) was excluded from the differentiation medium because it contains factors that improve the attachment of cells to the substrate [34], and was, therefore, a confounding variable. Although there was no significant difference in viability at earlier time points, by day 7 the viability of NG108-15 WT cells (70 ± 6%) was significantly lower (*p* = 0.0031) than that of NG108-15/Ngn2-L cells (94.2 ± 0.2%). Thus, Ngn2-induced differentiation reduced the likelihood of cell death compared with differentiation of the WT cell line (Figure 3B).

### 3.5. mRNA Sequencing Analysis of NG108-15/pBac_Ngn2-L Cells

In order to analyse any differences in the differentiation state between WT and Ngn2-L cells in more detail, total mRNAs were isolated and sequenced from cells that had been differentiated for 7 days. Sequencing results were analysed to identify variations in gene expression between Ngn2-L and WT NG108-15 cells. The mRNA sequencing data for general markers, neuronal progenitor markers and specific neuronal phenotypic markers were analysed using heat maps of log2 fold-change (log2FC) to investigate the change in gene expression of WT and Ngn2-L cells relative to undifferentiated controls. Direct counts of each mRNA per million reads (CPM) were also plotted for selected genes as a more quantitative statistical comparison of gene expression levels. The CPM for all cell lines for all expressed genes can be found in Table S1.

**Figure 4:**
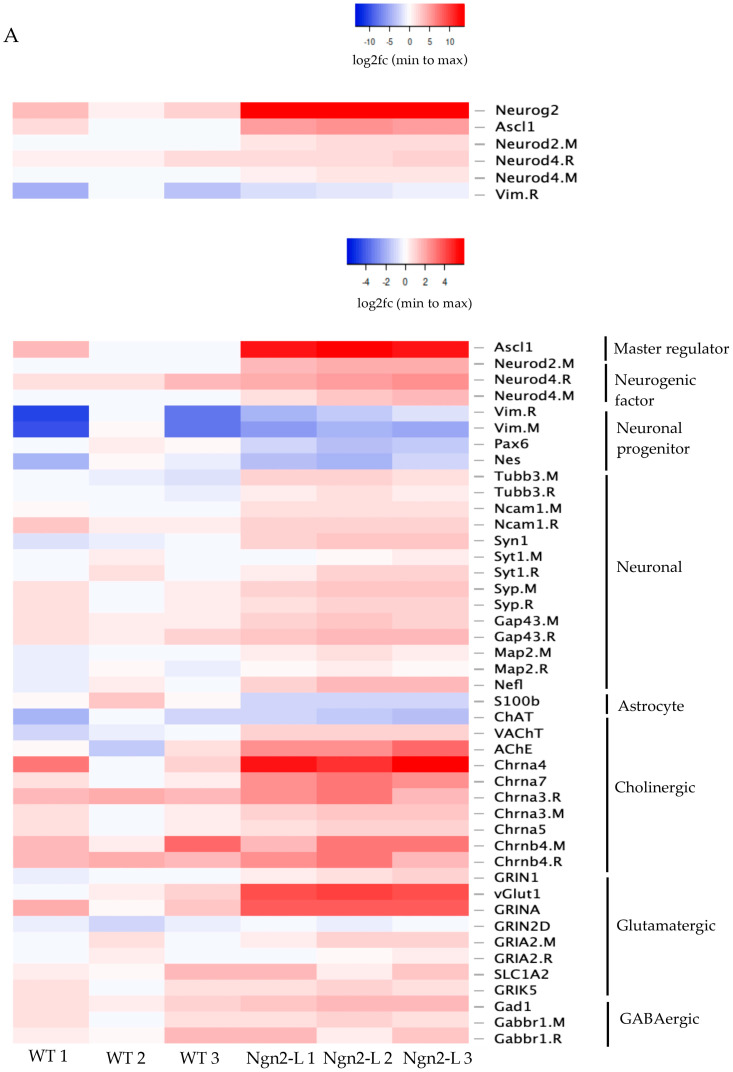

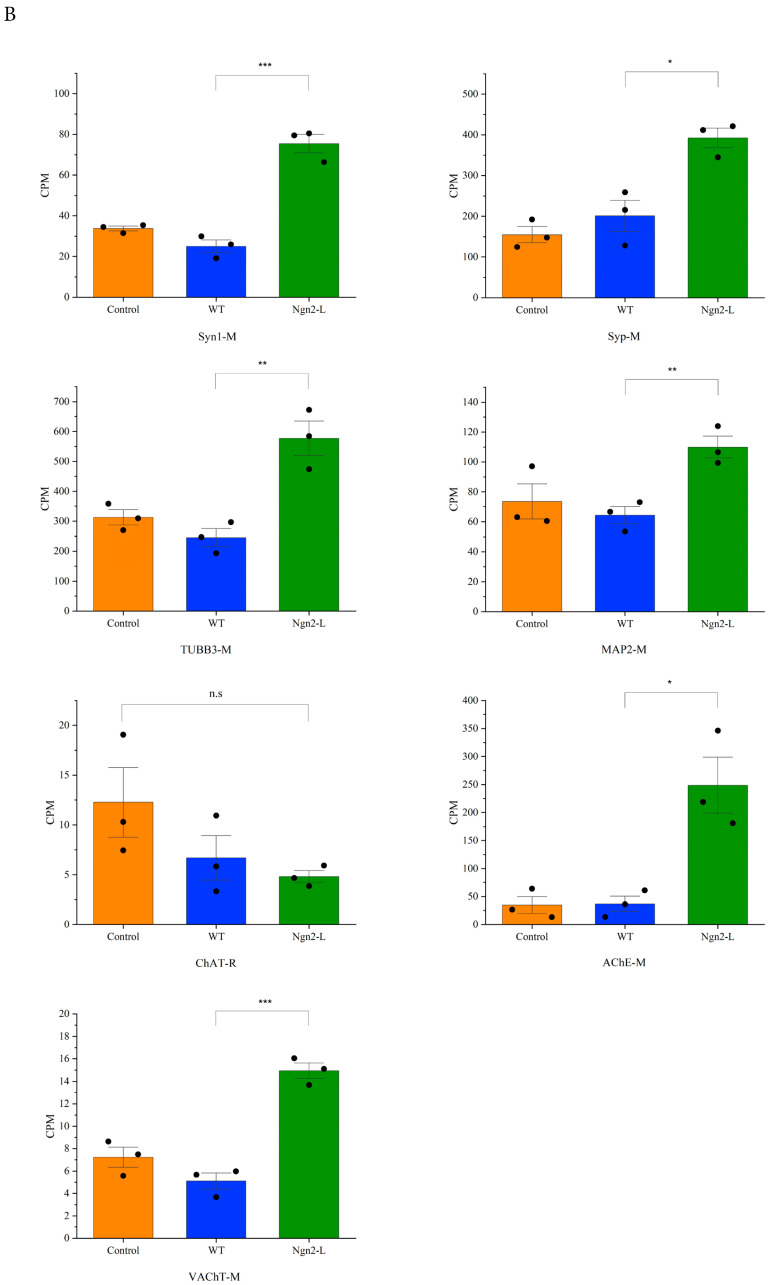

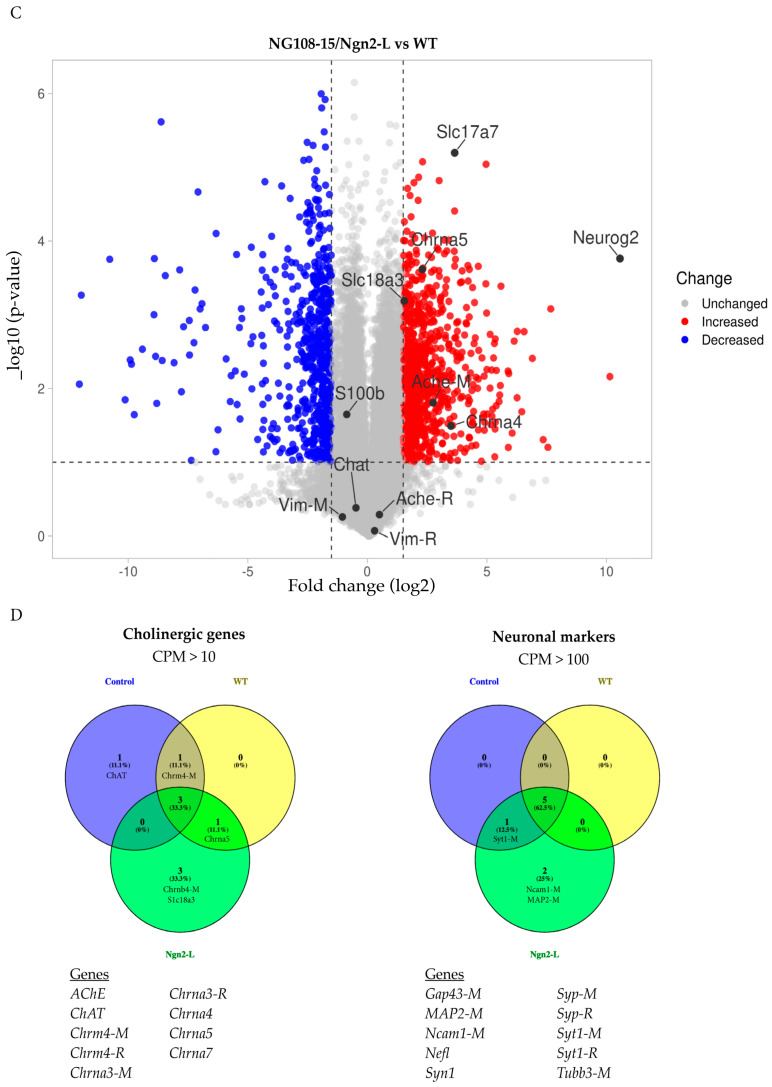

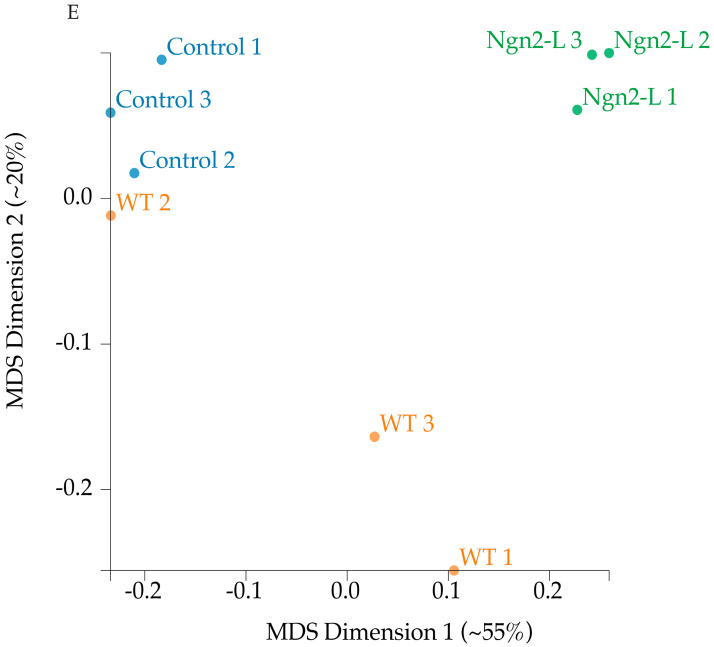
Gene expression changes after 7 days of differentiation in NG108-15 WT and Ngn2-L cells, relative to differentiated and undifferentiated WT controls. (**A**) Heatmaps summarising gene expression differences as log2-fold change (log2FC) of differentiated NG108-15 WT and Ngn2-L cells relative to undifferentiated WT control. (**B**) Average CPM of various genes including individual data points for each cell line replicate. Error bar represents SEM, *n* = 3 biological replicates, *** *p* = 0.0008, * *p* = 0.0134, ** *p* = 0.0069, ** *p* = 0.0080, * *p* = 0.0151, *** *p* = 0.0006, respectively. (**C**) Volcano plot showing, on the *X*-axis, the average gene expression (log2FC) of differentiated NG108-15 Ngn2-L cells compared to differentiated WT cells, with statistical significance (-Log10 *p*-value) on *Y*-axis. (**D**) Venn diagrams displaying cell types with relatively high expression of cholinergic genes and general neuronal markers. (**E**) Multi-dimensional scaling (MDS) plot displaying the primary and secondary dimensions in variance of gene expression (log2FC) for the various cell lines. n.s: not significant.

Log2FC values displayed in expression-based heatmaps, with triplicate experiments for each cell type, provide a comparison of several relevant genes relative to the undifferentiated control, as well as a comparison of all genes expressed (Figure 4A). Most genes were selected based on the expression profiles of NG108-15 cells, and the most commonly expressed neuronal markers, neuronal progenitor markers, and cholinergic markers. On the other hand, the genes selected for the glutamatergic and GABAergic markers were selected due to being key phenotypic markers for those neuronal cell types. The Log2FC values for the expression of these genes in the differentiated cell lines compared to the undifferentiated control can be found in Table S2.

As NG108-15 cells are a hybrid of mouse and rat cells, in some cases, both mouse and rat mRNA isotypes were detected, which were labelled M and R, respectively. As expected, Neurog2 (gene for Ngn2) was markedly and significantly upregulated in the NG108-15/Ngn2-L cells compared with WT cells due to the induction of the transgene. The log2FC for Neurog2 for the NG108-15/Ngn2-L was 13.3 ± 0.11, significantly greater than 2.4 ± 0.8 for WT cells (*p* = 0.0002). This very high log2FC value distorts the colour scale, so smaller changes are less apparent. Therefore, Neurog2 was included only in a heatmap with a few other genes (upper Figure 4A of neuronal genes) and excluded from the general heatmap (lower Figure 4A of neuronal genes), allowing for the relative expression of genes to be more readily visualised. The extent of Ngn2 expression changes relative to other genes is also apparent in the volcano plot (Figure 4C), where it shows as a clear outlier.

Further analysis of the log2FC data for the two differentiated cell lines relative to the undifferentiated control, in both sections of Figure 4A of neuronal genes, reveals a greater increase in the expression of neurogenic differentiation factors Neurod, Neurod2-M, and Neurod4 in Ngn2-L cells, compared with WT cells. Furthermore, there was a greater increase in the expression of the neuronal markers TUBB3, which encodes ßIII tubulin, microtubule-associated protein 2 (MAP2). A statistical comparison of the expression of these genes by CPM (Figure 4B) revealed that MAP2-M was expressed at 110 ± 7 CPM in the Ngn2-L cells, significantly higher (*p* = 0.0080) than 64 ± 6 CPM in WT cells. Moreover, TUBB3-M expression was 577 ± 58 CPM in the Ngn2-L cells, again significantly greater (*p* = 0.0069) than 245 ± 30 CPM in the WT cells (Figure 4B). Synaptic markers Synapsin I (Syn1) and Synaptophysin (Syp) also clearly displayed higher expression in differentiated Ngn2-L cells than in WT cells or undifferentiated controls (Figure 4A of neuronal genes). Syn1-M expression was 75 ± 5 CPM in Ngn2-L cells, significantly higher than 25 ± 3 CPM in WT cells (*p* = 0.0008). Likewise, Syp-M expression at 392 ± 24 CPM in the Ngn2-L cells was significantly greater than 201 ± 38 in WT cells (*p* = 0.0134). The volcano plot comparing the gene expression of the differentiated NG108-15/Ngn2-L cells compared to the differentiated WT cells indicated an increase in the Log2FC of various key genes, such as Neurog2, the vGlut1 gene Slc17a7, but also showed that ChAT was not increased in the NG108-15/Ngn2-L cells compared to the WT differentiated cells (Figure 4C). The Log2FC and -log10 *p* values for the expression of these genes in the differentiated NG108-15/Ngn2-L cells compared to the differentiated WT cells can be found in Table S3.

A Venn diagram of cells expressing neuronal markers above a certain threshold (Figure 4D) highlights that, whilst several markers are expressed in common, others are notably higher in Ngn2-L cells. Taken together, these findings support the notion that Ngn2-induced differentiation of NG108-15 cells enhances neuronal differentiation, generating more mature neuronal cells over this short period of 7 days.

Conversely, there was clearly a greater decrease in the astrocyte marker S100b in Ngn2-L cells than in WT cells. Additionally, there was a reduced expression of several neuronal progenitor genes including Nestin (NES) and Vimentin (VIM) in both cell lines, suggesting both cell lines had entered the differentiation pathway, beyond neural progenitors.

Relative to the general neuronal markers mentioned above, markers of more specific neuronal lineages showed greater increases in expression, over the undifferentiated control. In particular, these included key marker genes of a cholinergic phenotype, acetylcholinesterase (AChE), the enzyme secreted presynaptically and responsible for degrading ACh into choline and acetate in the synapse, and vesicular acetylcholine transporter (VAChT), responsible for packing ACh into synaptic vesicles and regulating cholinergic function [35]. The expression of AChE in Ngn2-L cells was 248 ± 50 CPM, significantly greater than 37 ± 14 in WT cells (*p* = 0.0151). Likewise, the expression of VAChT in Ngn2-L cells at 14.9 ± 0.7 CPM, was significantly higher than 5.1 + 0.7 CPM in WT cells (*p* = 0.0006). These increases in Ngn2-L cells, relative to WT cells, indicate that Ngn2 expression enhances differentiation to a cholinergic phenotype.

A notable exception to the enhanced expression of cholinergic genes was choline acetyltransferase (ChAT), which actually decreased relative to the undifferentiated control (12 ± 3 CPM), in both WT cells (6.7 ± 2.2 CPM) and Ngn2-L cells (4.8 ± 0.6 CPM) also being lower in Ngn2-L cells than WT cells, although none of these differences were statistically significant (Figure 4B). Interestingly, undifferentiated control cells expressed the highest CPM of ChAT compared with 6.70 ± 2.24 for NG108-15 WT cells and was lowest for the Ngn2-L cells (4.83 ± 0.61). However, the CPM was not statistically significant for control compared with WT cells (0.2506) or Ngn2-L cells (0.1035). Furthermore, the CPM values were quite low in all cases. These trends in ChAT expression differences appear contrary to the immunofluorescent staining in Figure 2C. There were also several nicotinic Ach receptor genes that showed increased expression, including alpha subunits (Chrna4, Chrna7, Chrna3), with Chrna4 expression being particularly high in Ngn2-L cells, and some beta subunits (Chrnb4-M, Chrnb5-R). Once again, a Venn diagram of cholinergic gene expression above a threshold (Figure 4D) highlights the higher expression of a set of cholinergic genes in Ngn2-L cells.

Interestingly, there was a pronounced increase in the expression of glutamatergic marker genes in Ngn2-L cells, particularly vGlut1, gene Slc17a7, (Figure 4A,C), which is required for concentrating vesicular glutamate for synaptic release. This indicates some redirection of differentiation by Ngn2 towards a glutamatergic phenotype. Despite this, when the NG108-15/Ngn2-L cells were stained to test for vGlut1 expression, the cells were vGlut1-negative (Figure 2D). Glutamate receptor related genes were also upregulated in Ngn2-L cells, notably ionotropic NDMA-Associated Protein 1 (GRINA), with much smaller increases in glutamate ionotropic receptor AMPA Type Subunit 2 (GRIA2), solute carrier family 1 member 2 (SLC1A2) and glutamate ionotropic receptor kainate type subunit 5 (GRIK5).

A multidimensional scaling (MDS) plot was generated to identify the main dimensions of variation in gene expression (log2FC) between the cell lines. As shown in Figure 4E, the cell lines were typically clustered in distinct regions on the plot. The Ngn2-L group showed a greater distance than WT cells from the undifferentiated control group in MDS Dimension 1. Conversely, the WT cells were clearly distinct in MDS Dimension 2 from both the control and Ngn2-L cells, with little difference between the last two. Interestingly, one of the WT cell samples is closer to the control group than to the other WT samples, indicating that the differentiation of WT cells is more heterogeneous than other cell samples. This heterogeneity is also apparent in Figure 4A heatmap of neuronal genes with one of the three samples being clearly distinct from the other two and showing less obvious differences to the control. This apparent variability may be due to the differentiation of WT cells being less consistent than for Ngn2-L cells, resulting in different rates or pathways of differentiation in different samples.

### 3.6. Analysis of Neuromuscular Junction Formation and Myotube Contractile Dynamics in NG108-15/C2C12 Co-Cultures

NG108-15 cells are known to differentiate into cholinergic neurons that behave like motor neurons. Co-culture of motor neurons with skeletal muscle cells is commonly used as a model of neuromuscular junction (NMJ) formation essential for voluntary muscle contraction. This process occurs when an action potential in the motor neuron leads to an influx of Ca^2+^ in the presynaptic terminal, stimulating ACh release, which binds to the AChRs on muscle cells, activating an action potential here that leads to muscle contraction [36]. In this study, NG108-15 cells were co-cultured with C2C12 myotubes to determine whether ectopic Ngn2 expression improves NMJ-like formation and spontaneous myotube contractions.

**Figure 5:**
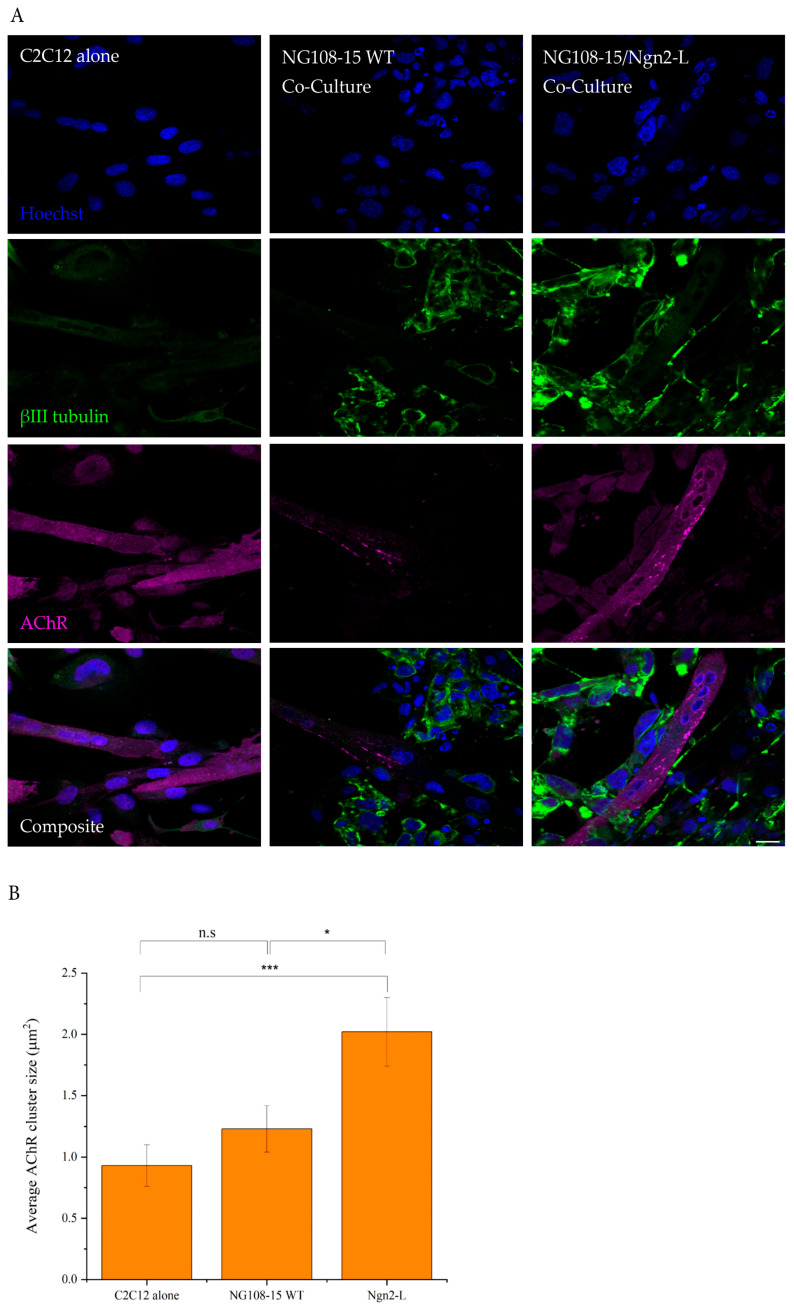
AChR cluster morphology in C2C12/NG108-15 co-cultures. (**A**) To investigate the formation of cholinergic synapses, fluorescent α-bungarotoxin was used to label AChRs and detect clusters, together with counter-staining with Hoechst to show nuclei and βIII-tubulin to stain Ng108-15-derived neurons. There was diffuse staining of AChRs in all cultures, but after co-culture, the AChR aggregates appeared larger. The AChRs were typically identified along the edges of the myotube and were most noticeable in Ngn2-L co-cultures. Magnification 60× Scale bar 20 μm. (**B**) Average AChR cluster size from image analysis was larger when the myotubes were co-cultured with WT NG108-15 cells compared with myotube monocultures, but this was not statistically significant. Conversely, AChR cluster size increased significantly when myotubes were co-cultured with NG108-15/Ngn2-L cells relative to either myotube monocultures or WT co-cultures, indicating a more robust NMJ-like formation. Results are presented as mean ± SEM, n = 10 images. * *p* = 0.26, *** *p* = 0.0039. n.s: not significant.

After 7 days of Ng108-15, C2C12 co-culture, the average size of the AChR aggregates in differentiated myotubes was assessed to measure NMJ formation. AChRs were expressed and clusters were present in myotubes from each group, including the myotube monocultures. Despite this, the aggregates were larger and more complex in the co-cultures (Figure 5A). The myotube cultures had some oval plaque-shaped AChR aggregates when cultured on their own, as described previously [37,38], whereas, cocultures showed more mature NMJs with some AChRs clusters being a more pretzel-like shape with enhanced clustering and localisation in areas with nerve cell contact, as reported earlier [38,39]. Again as seen previously [40], larger AChR clusters were observed at the edges of the myotubes in our myotube/neuronal co-cultures (Figure 5A).

Average AChR cluster size was quantified to compare the size of AChR aggregates between experimental groups (Figure 5B), showing that clusters were significantly larger for the NG108-15/Ngn2-L/myotube co-cultures (2.02 ± 0.28 μm^2^) than either myotubes cultured alone (0.93 ± 0.17 μm^2^, *p* = 0.0039) or myotubes cocultured with NG108-15-WT 1.23 ± 0.19 μm^2^, *p* < 0.05). Although AChR clusters appeared larger for NG108-15-WT/myotube co-cultures (1.23 ± 0.19 μm^2^) compared with myotubes cultured alone (0.93 ± 0.17 μm^2^), this was not statistically significant (*p* = 0.26). These results indicate that the co-culture of NG108-15/Ngn2-L cells with myotubes improves the formation of NMJ-like structures.

### 3.7. Quantification of Myotube Contractions

**Figure 6:**
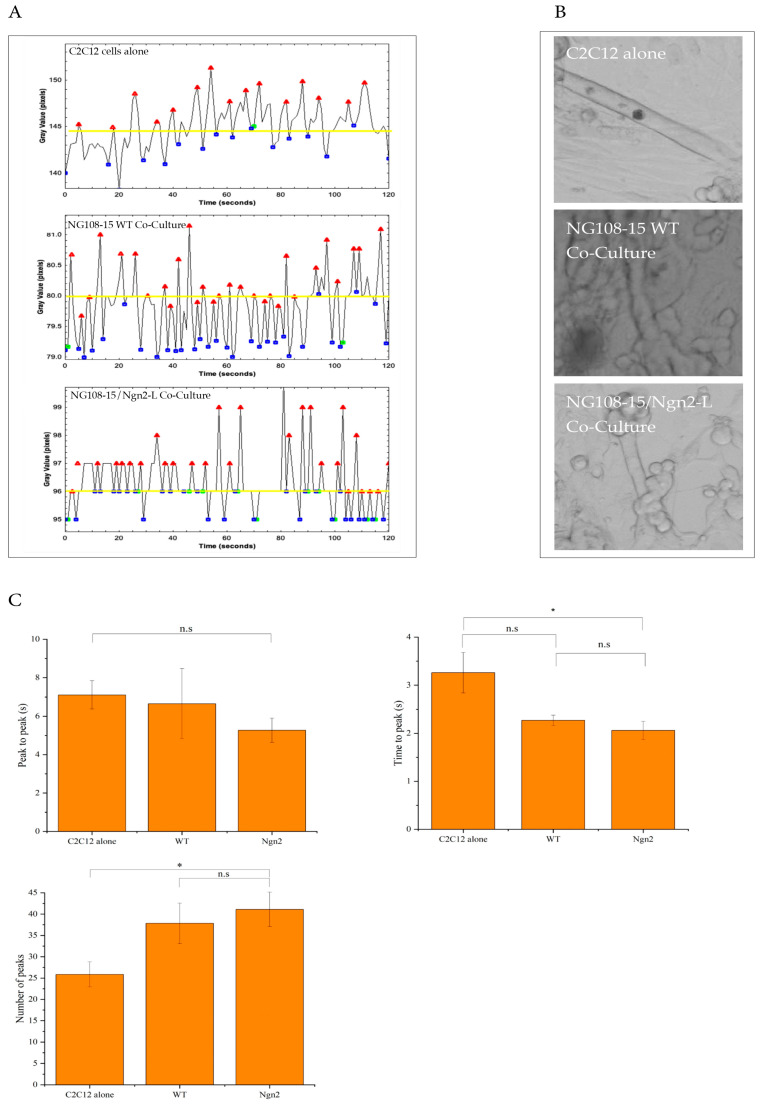
Quantification of myotube contractions in NG108-15/myotube co-cultures or single-cell myotube cultures. (**A**) Examples of plot profiles generated for myotube contractions in various conditions using Spiky toolkit. The plot profiles display peaks (red) and troughs (blue) and baseline parameters where registered peaks were excluded (yellow). (**B**) Examples of visual still-frame images of contracting myotubes used for analysis. Magnification 10× Scale bar 20 µm. (**C**) Peak to peak, average number of peaks and time to peak from spontaneous myotube contractions. The time between peaks (s) was slightly longer when myotubes were cultured alone compared with co-cultured with neuronal cells. The time taken to peak (s) was faster for myotubes in the neuronal co-cultures compared with myotubes cultured alone. Moreover, the time taken to peak was slightly higher in the WT co-cultures compared with the Ngn2-L co-cultures. The average number of peaks was significantly higher when the myotubes were co-cultured with Ngn2-L cells, than when cultured alone. There was no significant difference between the myotubes that were co-cultured with the Ngn2-L cells compared with the WT cells. Results are presented as mean, error bar represents SEM, C2C12 alone: *n* = 7 cells, NG108-15 WT: *n* = 6 cells, NG108-15/Ngn2-L *n* = 9 cells. Time to peak (s): * *p* = 0.014. Number of peaks: * *p* = 0.012. n.s: not significant.

C2C12-derived myotubes are capable of contracting spontaneously [24] as contractile proteins are expressed throughout differentiation. Spontaneous contractions were identified within 4–7 days of differentiation, although the likelihood of a contracting myotube reduced over time. As we predict that myotubes should be more likely to spontaneously contract in co-culture with neurons forming functional NMJs, we next analysed the functional dynamics of spontaneous myotube contraction using image-based methods. Time-series recordings of twitching myotubes were performed to measure distance changes between two select points on a myotube and investigate any effect on contractile dynamics of the co-culture of C2C12 cells with differentiated Ngn2-L or WT NG108-15 cells. An example of plot profiles and images generated for twitching myotubes is shown in Figure 6A. Myotube contractions were analysed using the Spiky toolkit (Spiky v0.52 ImageJ Toolkit), which, although originally designed for cardiac muscle and cardiomyocyte analysis, could also accurately detect and analyse the peaks from the myotube-derived kymographs.

The time between myotubes contraction peaks (Figure 6C) was not significantly different, whether they were cultured alone (7.1 ± 0.7 s) or co-cultured with NG108-15 WT cells (6.6 ± 1.8 s) or Ngn2-L cells (5.3 ± 0.6 s). The average number of peaks per 200 s recording window was higher when the myotubes were co-cultured with neuronal cells (Figure 6C). The average number of twitches for the myotubes cultured alone (*n* = 7 cells) was 25 ± 3, which increased when co-cultured with NG108-15 WT cells (*n* = 6 cells) to 37.83 ± 4.74, although this apparent increase was not significant. The number of twitches, or peaks, increased further for the NG108-15/Ngn2-L co-cultures (*n* = 9 cells) to 41 ± 4, which was significantly greater (*p* = 0.012) than for myotubes cultured alone. There was no significant difference in the number of peaks when myotubes were co-cultured with the NG108-15 WT, compared with NG108-15/Ngn2-L cells (*p* = 0.61).

The time taken to peak for the myotubes cultured alone was 3.26 ± 0.42 s, which fell for NG108-15 WT cells (2.27 ± 0.11 s), and was lowest for the NG108-15/Ngn2-L co-cultures (2.06 ± 0.19 s). Although the difference in time taken to peak was approaching significance in the WT co-cultures compared with myotubes cultured alone (*p* = 0.058), this was significantly different in the Ngn2-L co-cultures compared with myotubes cultured alone (*p* = 0.014).

In summary, the results show that the addition of neuronal cells improved the functional dynamics of myotubes with the number of peaks decreasing and time taken for the myotube to reach each peak decreasing with the addition of NG108-15/Ngn2-L cells, relative to myotubes cultured alone. These parameters were not significantly different in NG108-15 WT co-cultures, compared with myotubes cultured alone. The findings confirm that the NG108-15/Ngn2 neuronal cells improve the functional differentiation outcomes for myotubes.

## 4. Discussion

We stably transfected the NG108-15 cell line with Ngn2 by pBac transposition and performed doxycycline-induced neuronal differentiation to test whether the transcription factor improved differentiation of the cell line. Initially, cell pools were selected via FACS for different ranges of fluorescence intensity, expected to correlate with Ngn2 expression. These pools were then tested for the effect of Ngn2 induction on differentiation. We then evaluated morphology and viability of the cell line post-differentiation, moving onto molecular characterisation by immunocytochemistry and mRNA sequencing. Lastly, in co-culture with C2C12-derived myotubes, we tested the functional capacity of the NG108-15/Ngn2-L cell line to improve NMJ formation, AChR clustering, and myotube contractile dynamics.

In recent decades, the ready availability of stem cells, from a variety of sources, and iPS cells have provided a reliable platform for the development of in vitro differentiation protocols. More recently, the forced or ectopic expression of transcription factors in these cell types has enhanced the speed and reliability of differentiation. Notably, Ngn2 expression in stem cells and iPS cells has been repeatedly demonstrated to have a pro-neural effect on differentiation [7]. In many cases, expression of Ngn2 alone directs differentiation towards glutamatergic neuronal lineages [8,9,10] but, depending on the original state of the cells, co-expression of other transcription factors or other conditions, differentiation to a variety of neuronal lineages is possible [7,10]. Despite these developments, the more traditional use of immortalised cell lines as in vitro differentiation models remains a viable alternative, due to their relatively simple culture and differentiation conditions and consistent outcomes.

Here, we tested the hypothesis that the addition of ectopic Ngn2 expression to the well-characterised NG108-15 immortalised neuronal cell line, would enhance differentiation to its regular cholinergic phenotype and potentially redirect towards a glutamatergic lineage, as often seen with the ectopic expression of Ngn2. Following seven days of Ngn2-induced differentiation, we found that Ngn2 improved neurite elongation and cell viability. Additionally, mRNA sequencing analysis revealed elevated expression of neuronal and synaptic markers (MAP2, Syn-1, Syp, TUBB3) in Ngn2-L cells compared with WT, suggesting accelerated neuronal maturation. While ChAT protein was detected initially, mRNA sequencing analysis showed upregulation of cholinergic markers AChE and VAChT, but not ChAT mRNA, indicating differentiation might need to be extended. vGlut1 expression suggested the potential for glutamatergic differentiation.

Ectopic expression of TFs, such as Ngn2, in stem cells is now a well-established approach for driving differentiation to specific cell lineages, such as glutamatergic neurons. The novelty of the research presented here is applying this approach to an immortalised neuronal cell line. A similar approach has been carried out by Grubišić and colleagues on an immortalised muscle cell line, C2C12, where ectopic expression of transcription factors E12 and MyoD was capable of inducing myotube differentiation, in what would have been considered non-differentiation conditions [41]. It was found that MyoD induced the fusion of myoblasts into myotubes and the elongation of muscle fibres, demonstrating, in a different cell type to the research presented here, that transcription factors are capable of inducing enhanced differentiation of cell lines to their default phenotype. These results are consistent with the current work, demonstrating the ability of Ngn2 to induce differentiation of the NG108-15 cell line to its default cholinergic phenotype. We also showed here that, along with enhanced cholinergic differentiation, Ngn2 increased the expression of markers for glutamatergic neurons, consistent with the ability of Ngn2 to rapidly induce hiPSCs into glutamatergic neurons positive for vGlut1 [7]. Conversely, the ability of Ngn2 to give rise to cholinergic neurons from human fibroblasts [10] is somewhat similar to our findings in the NG108-15 cell line, suggesting that Ngn2 can drive differentiation to various neuronal subtypes that may depend on the differentiation status of the starting cells.

Co-culture of the NG108-15/Ngn2-L cells with C2C12 cells demonstrated increased cluster size and enhanced myotube contractions by increasing the number of peaks and reducing peak times compared with WT, implying faster cholinergic synapse formation. The improvements to myotube dynamics suggest an improved model of NMJ formation, which is beneficial for identifying drugs or therapeutics for treating specific neuromuscular deficits. Because it is difficult to investigate NMJs using animal models due to physiological relevance, it is important to model NMJs as effectively as possible. Electrophysiological measures would provide further validation.

## 5. Conclusions

This study investigated the potential for the transcription factor Ngn2 to enhance the neuronal differentiation of NG108-15 cells. Ngn2 induction caused robust neurite elongation, network formation, and improved cell viability and morphology. Moreover, co-culture with C2C12 cells revealed an improvement in NMJ-like formations and enhanced spontaneous contraction, although this has yet to be functionally validated by electrophysiology and activated contractions. Taken together, these findings suggest that Ngn2 improves neuronal differentiation and motor neuron modelling potential without altering the default phenotype. These findings offer promise for future research because improved models of NMJs can help improve our understanding of NMJ development and function, providing insight into neuromuscular diseases, accelerate drug discovery and improve the accuracy of therapeutic screenings, offering greater promise for treating neuromuscular disorders.

## Data Availability

The original contributions presented in this study are included in the article. Further inquiries can be directed to the corresponding authors.

## Supplementary Materials

The following supporting information can be downloaded at: https://www.mdpi.com/article/10.3390/biom15050637/s1, Table S1: mRNA sequencing CPM data for all cell lines. Table S2: Heatmap data of log2FC of differentiated cell lines compared to undifferentiated control—All genes. Table S3: Volcano Plot of differentiated NG108-15 Ngn2-L cells relative to WT cells. Figure S1: Heatmap of log2FC of all genes of differentiated cell lines relative to undifferentiated control.
