# Correction: Meli et al. Ngn2-Induced Differentiation of the NG108-15 Cell Line Enhances Motor Neuronal Differentiation and Neuromuscular Junction Formation. *Biomolecules* 2025, *15*, 637

**DOI:** 10.3390/biom15121720

**Published:** 2025-12-11

**Authors:** Madeline Meli, Kristy Swiderski, Jinchao Gu, Ben Rollo, Ben Bartlett, Marissa K. Caldow, Gordon S. Lynch, Patrick Kwan, Huseyin Sumer, Brett A. Cromer

**Affiliations:** 1 Department of Chemistry and Biotechnology, School of Science, Computing and Engineering Technologies, Swinburne University of Technology, Melbourne, VIC 3122, Australia; 2 Centre for Muscle Research, Department of Anatomy and Physiology, School of Biomedical Sciences, Faculty of Medicine, Dentistry and Health Sciences, The University of Melbourne, Melbourne, VIC 3122, Australiagsl@unimelb.edu.au (G.S.L.); 3 Department of Neuroscience, Central Clinical School, Monash University, Alfred Centre, Melbourne, VIC 3122, Australia

There was an error in the original publication [1]. Several references were numbered incorrectly in the text due to a software error when compiling the reference list. References [20–24] are in wrong order. The correct reference order appears below, with this correction, the order of some references has been adjusted accordingly.

20. QIAGEN QIAGEN RNeasy(R) Mini Handbook. Available online: https://www.qiagen.com/us/resources/download.aspx?id=f646813a-efbb-4672-9ae3-e665b3045b2b&lang=en (accessed on 9 May 2024).
21. Oliveros, J.C. Venny. An Interactive Tool for Comparing Lists with Venn’s Diagrams. Available online: https://bioinfogp.cnb.csic.es/tools/venny/index.html (accessed on 12 April 2025).
22. Babicki, S.; Arndt, D.; Marcu, A.; Liang, Y.; Grant, J.R.; Maciejewski, A.; Wishart, D.S. Heatmapper: Web-enabled heat mapping for all. *Nucleic Acids Res.* **2016**, *44*, 147–153.
23. Goedhart, J.; Luijsterburg, M.S. VolcaNoseR is a web app for creating, exploring, labeling and sharing volcano plots. *Sci. Rep.* **2020**, *10*, 20560.
24. Hong, W.S. Microsystems Engineering Assisted Cell and Tissue Culture Models: Detection and Therapeutic Applications of Botulinum Neurotoxin Type A. Ph.D. Thesis, University of Wisconsin–Madison, Madison, WI, USA, 2015.

Additionally, the author’s name “Brett Cromer” has been updated to “Brett A. Cromer” to reflect the author’s full name.

The authors state that the scientific conclusions are unaffected. This correction was approved by the Academic Editor. The original publication has also been updated.
