# Supplementary figures and images for: Ngn2-Induced Differentiation of the NG108-15 Cell Line Enhances Motor Neuronal Differentiation and Neuromuscular Junction Formation
Source: Biomolecules. 2025 Apr 29;15(5):637. doi: 10.3390/biom15050637 (PMC12108711; doi:10.3390/biom15050637)

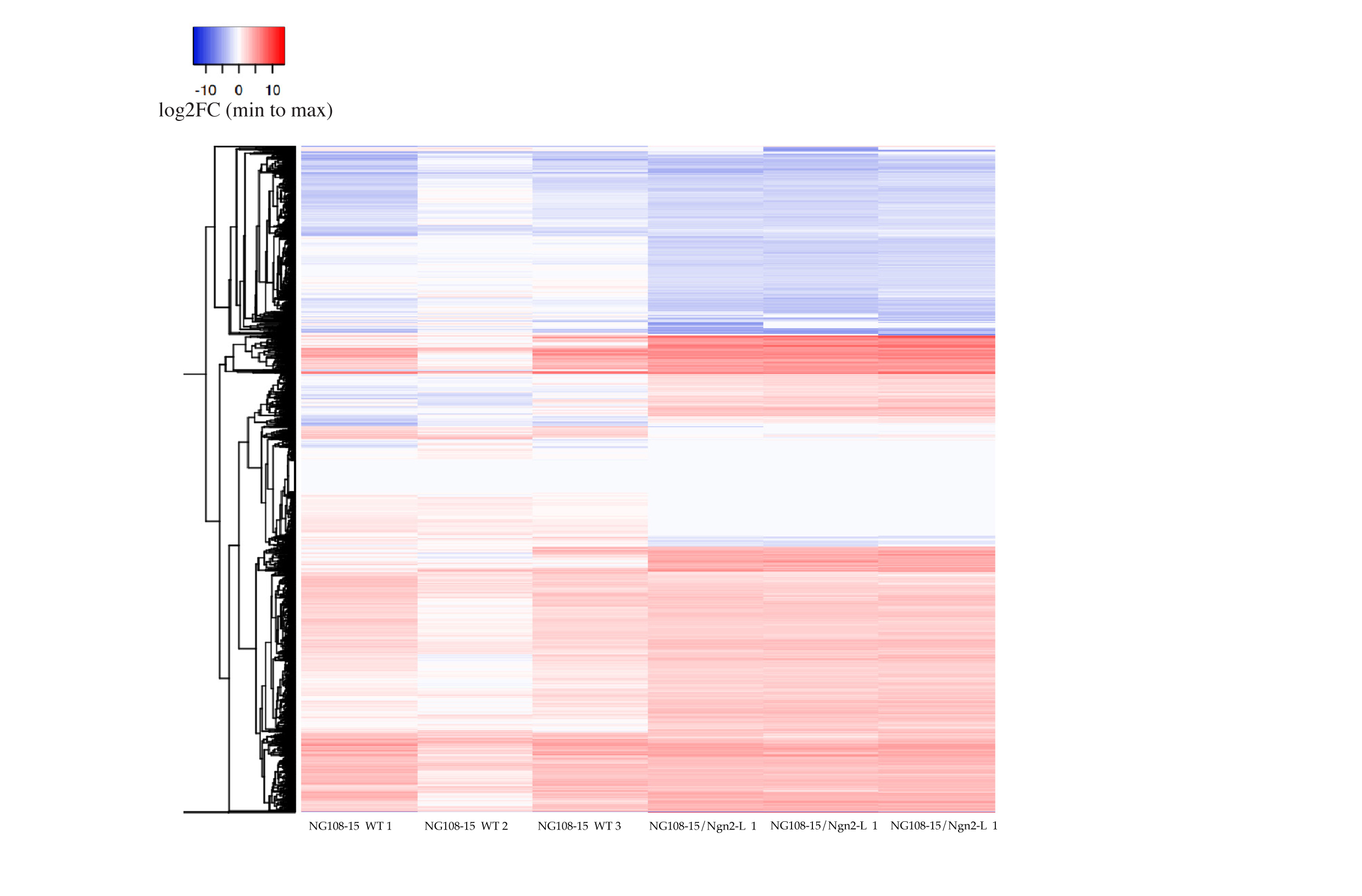

Supplement: Supplementary file 1 [file biomolecules-15-00637-s001.zip › biomolecules-3574371 Figure S1.jpg]
